# How many inpatients in our hospitals have foot complications? The Foot Disease in Inpatients Study

**DOI:** 10.1186/1757-1146-8-S2-O25

**Published:** 2015-09-22

**Authors:** Peter A Lazzarini, Vanessa Ng, Suzanne S Kuys, Maarten C Kamp, Michael C d'Emden, Courtney Thomas, Jude Wills, Ewan M Kinnear, Scott Jen, Sheree E Hurn, Lloyd Reed

**Affiliations:** 1 School of Clinical Sciences, Queensland University of Technology, Brisbane, Queensland, 4059, Australia; 2 Allied Health Research Collaborative, Metro North Hospital & Health Service, Queensland Health, Brisbane, Queensland, 4032, Australia; 3 Department of Podiatry, Metro North Hospital & Health Service, Queensland Health, Brisbane, Queensland, 4032, Australia; 4 Musculoskeletal Research Program, Griffith Health Institute, Griffith University, Gold Coast, Queensland, 4222, Australia; 5 Department of Endocrinology and Diabetes, Royal Brisbane and Womens Hospital, Brisbane, Queensland, 4029, Australia; 6 School of Medicine, The University of Queensland, Brisbane, Queensland, 4072, Australia; 7 Department of Podiatry, North West Hospital & Health Service, Mount Isa, Queensland, 4825, Australia; 8 Department of Podiatry, Central Queensland Hospital & Health Service, Rockhampton, Queensland, 4700, Australia; 9 Department of Podiatry, West Moreton Hospital & Health Service, Queensland Health, Ipswich, Queensland, 4305, Australia

**Keywords:** Foot, inpatients, disease, complications, prevalence

## Background

Foot complications have been found to affect large proportions of hospital in patients with diabetes. However, no studies have investigated the proportion of foot complications affecting all people in general inpatient populations. The aims of this cross-sectional study were to investigate the point-prevalence of different foot complications in general inpatient populations, analyse differences in diabetes and non-diabetes sub-groups, and examine characteristics of people primarily admitted for a foot complication.

## Methods

Eligible participants were all adults admitted overnight, for any reason, into five diverse hospitals on one day; excluding maternity, mental health and cognitively impaired patients. All participants underwent a physical foot examination, by trained podiatrists using validated measures, to clinically diagnose different foot complications; including foot wounds, infections, deformity, peripheral arterial disease (PAD) and peripheral neuropathy (PN). Data were also collected on participants' primary reason for admission and a range of demographic, social determinant, medical history, foot complication history, self-care and footwear risk factors.

## Results

Overall, 733 participants consented (83% of eligible participants); mean(±SD) age 62(±19) years, 480 (55.8%) male and 172 (23.5%) had diabetes. Foot complication prevalence included: wounds 9.0% (95% CI) (5.1-8.7), infections 3.3% (2.2-4.9), deformity 22.4% (19.5-26.7), PAD 21.0% (18.2-24.1) and PN 22.0% (19.1-25.1). Diabetes populations had significantly more foot complications than non-diabetes (*p* < 0.01); wounds (15.7% vs 7.0%), infections (7.1% vs 2.2%), deformity (30.5% vs 19.9%), PAD (35.1% vs 16.7%) and PN (43.3% vs 15.4%). Foot complications were the primary reason for admission in 7.4% (95% CI) (5.7-9.5) of all participants. In a backwards stepwise multivariate analysis having a foot complication as the primary reason for admission was independently associated (OR (95% CI) with foot wounds (18.9 (7.3-48.7)), foot infections (6.0 (1.6-22.4)), history of amputation (4.7 (1.3-17.0) and PAD (2.9 (1.3-6.6)).

## Conclusions

Findings of this study indicate one in every ten hospital inpatients had an active foot wound or infection. In patients with diabetes had significantly higher proportions of foot complications than non-diabetes inpatients. Remarkably one in every thirteen inpatients in this study were primarily hospitalised for a foot complication. Further research and policy is required to tackle this seemingly large inpatient foot complication burden.

